# The PEritoneal SUrface CAlculator (PESUCA): A new tool to quantify the resected peritoneal surface area after cytoreductive surgery

**DOI:** 10.1515/pp-2019-0031

**Published:** 2020-02-26

**Authors:** Philipp Schredl, Jan Philipp Ramspott, Daniel Neureiter, Klaus Emmanuel, Tarkan Jäger

**Affiliations:** Department of Surgery, Paracelsus Medical University, Salzburger Landeskliniken (SALK), Müllner Hauptstraße 48, Salzburg, Austria; Department of Gynecology and Gynecologic Oncology, Kliniken Essen-Mitte, Henricistraße 92, Essen, Germany; Institute of Pathology, Paracelsus Medical University, Salzburger Landeskliniken (SALK), Müllner Hauptstr. 48, Salzburg, Austria; Department of Surgery, Paracelsus Medical University, Salzburger Landeskliniken (SALK), Müllner Hauptstraße 48, Salzburg, Austria

**Keywords:** HIPEC, intraperitoneal drug dosing, PCI, peritoneal surface malignancies, peritonectomy

## Abstract

**Background:**

The body surface area (BSA) is taken as a measure for the effective contact area for dosing in hyperthermic intraperitoneal chemotherapy (HIPEC). Currently, the pharmacokinetic effect of the reduced peritoneal surface area (PSA) after cytoreductive surgery (CRS) during HIPEC remains unclear. Here a proprietary software solution (PEritoneal SUrface CAlculator (PESUCA)) to quantify the resected PSA in patients with peritoneal surface malignancies (PSM) undergoing CRS and HIPEC is presented.

**Methods:**

The PESUCA tool was programmed as a desktop and online software solution. The applicability was evaluated in 36 patients. The programming-algorithm is briefly summarized as follows: (1) calculation of BSA, (2) correlation to PSA, (3) calculation of the relative proportion of 40 different anatomical regions to total PSA before CRS, (4) instantaneous input of each resected proportion in the 40 anatomical regions during CRS, and (5) determination of the resected and remaining PSA after CRS.

**Results:**

The proof of concept revealed a mean PSA of all patients before CRS of 18,741 ± 321 cm^2^ compared to 13,611 ± 485 cm^2^ after CRS (p<0.0001). Patients’ supramesocolic and inframesocolic visceral and parietal peritoneal area before and after CRS procedure were quantitatively determined.

**Conclusions:**

Here the first tool that enables detailed PSA quantification in patients with PSM undergoing CRS is presented. This makes the software a valuable contribution to ensue more accurate assessment and improved comparability of peritoneal disease extent. Furthermore, after external validation, PESUCA could be the basis for dose adjustment of intraperitoneal chemotherapy regimens based on the remaining PSA after CRS.

## Introduction

During the last two decades, new treatment protocols that combine cytoreductive surgery (CRS) and perioperative hyperthermic intraperitoneal chemotherapy (HIPEC) for patients suffering from peritoneal surface malignancies (PSM) were developed. Heated chemotherapeutic drugs are used locally, thereby reducing systemic toxicity [1, 2]. Since HIPEC procedures were first developed in the 1980s, multiple studies have been conducted resulting in widespread discussions about its real benefit and associated patients’ risks. Recently, these discussions were readdressed by the Prodige 7 trial [3] and the Dutch ovarian cancer HIPEC study [4]. Despite promising results showing its efficacy in the treatment of abdominal and pelvic malignancy, there is no standardized protocol for the use of HIPEC. Eight parameters affecting HIPEC efficacy are described so far: choice of chemotherapeutic agent, carrier solution, dosing regimen, perfusate volume, temperature, procedure duration, delivery technique, and adequate patient selection [5, 6]. An important controversial issue is the choice of chemotherapeutic dosing regimen. Within the context of HIPEC, a dose is the amount of a drug administered at one specific time whereas dosage means the amount and rate of administration (time frequency) of a certain substance. A drug concentration is the amount of a substance per defined space. Currently, two dosing regimens are applied. Most centers use body surface area (BSA) (mg/m^2^) (in a similar fashion to systemic chemotherapy) to determine the dose of chemotherapy, but concentration-based protocols are also applied [7].

In BSA-based protocols, fixed doses (mg/m^2^) are diluted in different volumes of the carrier solution leading to different drug concentrations. Varying volumes are caused by several factors (e. g. patient’s body composition and HIPEC delivery techniques). In contrast, concentration-based protocols with BSA-based drug doses and BSA-based or absolute volumes of carrier solution result in fixed drug concentrations [7, 8].

Regardless of the method used to calculate the dose (BSA- vs. concentration-based), the remaining PSA after CRS is not considered. The two-compartment Dedrick model of intraperitoneal chemotherapy is an application of Fick’s law of diffusion. It describes the transfer of a drug from the peritoneal cavity to the body compartment (blood): rate of mass transfer=PA (*C*_P_ – *C*_B_), where PA is permeability area (PA=effective peritoneal contact area, *A* × permeability, *P*), *C*_P_ is the concentration in the peritoneal cavity, and *C*_B_ is the concentration in the blood [9]. The size of the effective contact area of the peritoneum and the drug concentration are the most important components in this formula. Thus, the remaining intraperitoneal chemotherapy concentration depends upon the PSA.

Until now, it has not been possible to quantify the resected and the remaining PSA in patients undergoing CRS. Here, the applicability of the PEritoneal SUrface CAlculator (PESUCA) tool to quantify the PSA in 36 patients with PSM before and after CRS is presented.

## Materials and methods

### PESUCA tool

Microsoft^®^ Access^®^ with Visual Basic for Applications was used to program the desktop version of PESUCA, whereas the online version (https://pesuca.net/) was programmed with Python™ (https://www.python.org/). According to Albanese et al., the total PSA in the software consists of four groups: (1) supramesocolic visceral peritoneum (SMCVP), (2) supramesocolic parietal peritoneum (SMCPP), (3) inframesocolic visceral peritoneum (IMCVP), and (4) inframesocolic parietal peritoneum (IMCPP). Each group includes different peritoneal regions (SMCVP: 16 areas, SMCPP: 6 areas, IMCVP: 12 areas, and IMCPP: 6 areas; total: 40) (Table 1) [10]. The percentage contribution of each peritoneal region in relation to the total PSA before CRS was determined [10]. All 40 regions were included into the software as input fields with the possibility to be filled in by numbers ranging from 0% to 100%. Based on the DuBois and DuBois formula [11], PESUCA was programmed to determine the BSA (cm^2^) using height (cm) and weight (kg) data.

**Table 1:** Anatomical peritoneal regions inserted in the PESUCA tool (based on [10]).

No.	Supracolic peritoneum, visceral area	Supracolic peritoneum, parietal area	Infracolic peritoneum, visceral area	Infracolic peritoneum, parietal area
1	Liver	Right diaphragmatic wall	Mesentery	Right antero-lateral infraumbilical wall
2	Gastrocolic ligament	Left diaphragmatic wall	Jejunum-ileum	Left antero-lateral infraumbilical wall
3	Stomach	Right antero-lateral supraumbilical wall	Greater omentum	Left dorsal infracolic parietal wall
4	Spleen	Left antero-lateral supraumbilical wall	Sigmoid colon	Right dorsal infracolic parietal wall
5	Transverse mesocolon: superior layer	Right dorsal supracolic parietal wall	Transverse colon	Left lateral pelvic wall
6	Lesser omentum	Left dorsal supracolic parietal wall	Transverse mesocolon: inferior layer	Right lateral pelvic wall
7	Falciform ligament		Caecum v. appendix ascending colon	
8	Pancreas		Sigmoid mesocolon	
9	Gastrosplenic ligament		Uterus and broad ligaments	
10	Teres ligament		Rectum	
11	Duodenum		Descending colon	
12	Left triangular ligament		Urinary bladder	
13	Gall bladder			
14	Lienorenal ligament			
15	Right triangular ligament			
16	Abdominal esophagus			

PESUCA equates BSA with PSA if no values are inserted in any of the 40 anatomical regions according to Albanese et al., who reported that the PSA can be estimated from BSA formulas [10]. Depending on the inserted values inputted into the 40 different anatomical regions, PESUCA calculates the PSA in cm^2^. This is obtained by subtracting the inserted numbers from the total PSA. PESUCA was programmed with the following formulas:

1. Calculation of BSA (m^2^)BSA (m^2^) = 0.20247 × height (m)^0.725^ × weight (kg)^0.425^ [11]
2. Conversion of (m^2^) in (cm^2^)BSA (m^2^) x 10,000 = BSA (cm^2^)
3. Correlation of BSA (cm^2^) and PSA (cm^2^) (according to [10])BSA (cm^2^) = PSA (cm^2^)
4. Definition of total PSA (100%)PSA (cm^2^) = 100%
5. Fixed percentage contributions of 40 anatomical regions (X_1_–X_40_%) to the total PSA (cm^2^) (according to [10])X_1-40_% of total PSA = X_1-40_
6. Assignment of fixed percentage contributions of 40 anatomical regions (X_1-40_%) to absolute PSA values (cm^2^)[(PSA (cm^2^)/100%)] × X_1-40_%
7. Calculation of the individual PSA_before CRS_ as the sum of (6.)PSA_before CRS_ (cm^2^) = sum of [(PSA (cm^2^)/100%)] × X_1-40_%
8. Assignment of individual percentage contributions of 40 resected anatomical regions (Y_1-40_%) to absolute PSA values (cm^2^)[(PSA (cm^2^)/100%)] × Y_1-40_%
9. Calculation of the individual PSA_after CRS_ as the sum of (8.)PSA_after CRS_ (cm^2^) = sum of [(PSA (cm^2^)/100%)] × Y_1-40_%
10. Resected PSA (cm^2^)PSA_resected_ (cm^2^) = PSA_before CRS_ (cm^2^) – PSA_after CRS_ (cm^2^)
11. Peritoneal surface ratio before CRS (%): [PSA_before CRS_ (cm^2^)/BSA (cm^2^)]/100
12. Peritoneal surface ratio after CRS (%): [PSA_after CRS_ (cm^2^)/BSA (cm^2^)]/100X1-40 Representation of 40 anatomical regions before CRS in patientsY1-40 Representation of 40 anatomical regions after CRS in patients

PESUCA was used to calculate the PSA in 36 patients with PSM undergoing CRS and HIPEC. Before CRS was started, weight and height data of the patients were entered in the tool. During CRS, the assistant in the operating room instantaneously entered the amount of the resected peritoneal area (%) in the corresponding 40 anatomical regions. Here, 0% means no peritoneal resection, 100% means complete peritoneal resection. Thus, higher numbers show higher peritonectomy extent in the appropriate anatomical region.

### Statistical analysis

Continuous variables were expressed as mean ± SD after checking normality of the differences with the Shapiro–Wilk test. Differences in PSA before and after CRS were analyzed by the unpaired *t*-test. All tests were two-sided and p values<0.05 were considered statistically significant. All statistical analyses in this report were performed using STATA (StataCorp. 2015. StataStatistical Software: Release 14. College Station, TX, USA, Stata-Corp LP) and GraphPad Prism version 6.00 for Windows, GraphPad Software, La Jolla, California, USA, www.graphpad.com.

### Compliance with ethical standards

All procedures performed in this study were in accordance with the ethical standards of the institutional and/or national research committee and with the 1964 Helsinki declaration and its later amendments or comparable ethical standards.

## Results

**Figure 1:**
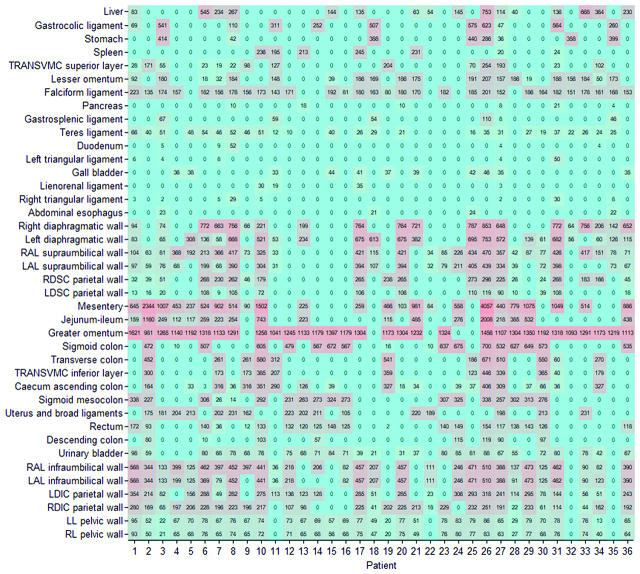
Resected peritoneal surface area (cm^2^) heat map. All patients who underwent CRS and HIPEC are shown. Patient numbers are labeled on the *x*-axis, and peritoneal areas according to [10] are labeled on the *y*-axis. Darker colors indicate larger peritoneal surface resections and numbers indicate absolute resected peritoneal surface areas in (cm^2^) calculated by PESUCA. TRANSVMC=transverse mesocolon, RAL=Right antero-lateral, LAL=left antero-lateral, RDSC=right dorsal supracolic, LDSC=left dorsal supracolic, LDIC=left dorsal infracolic, RDIC=right dorsal infracolic, LL=left lateral, RL=right lateral.

**Figure 2:**
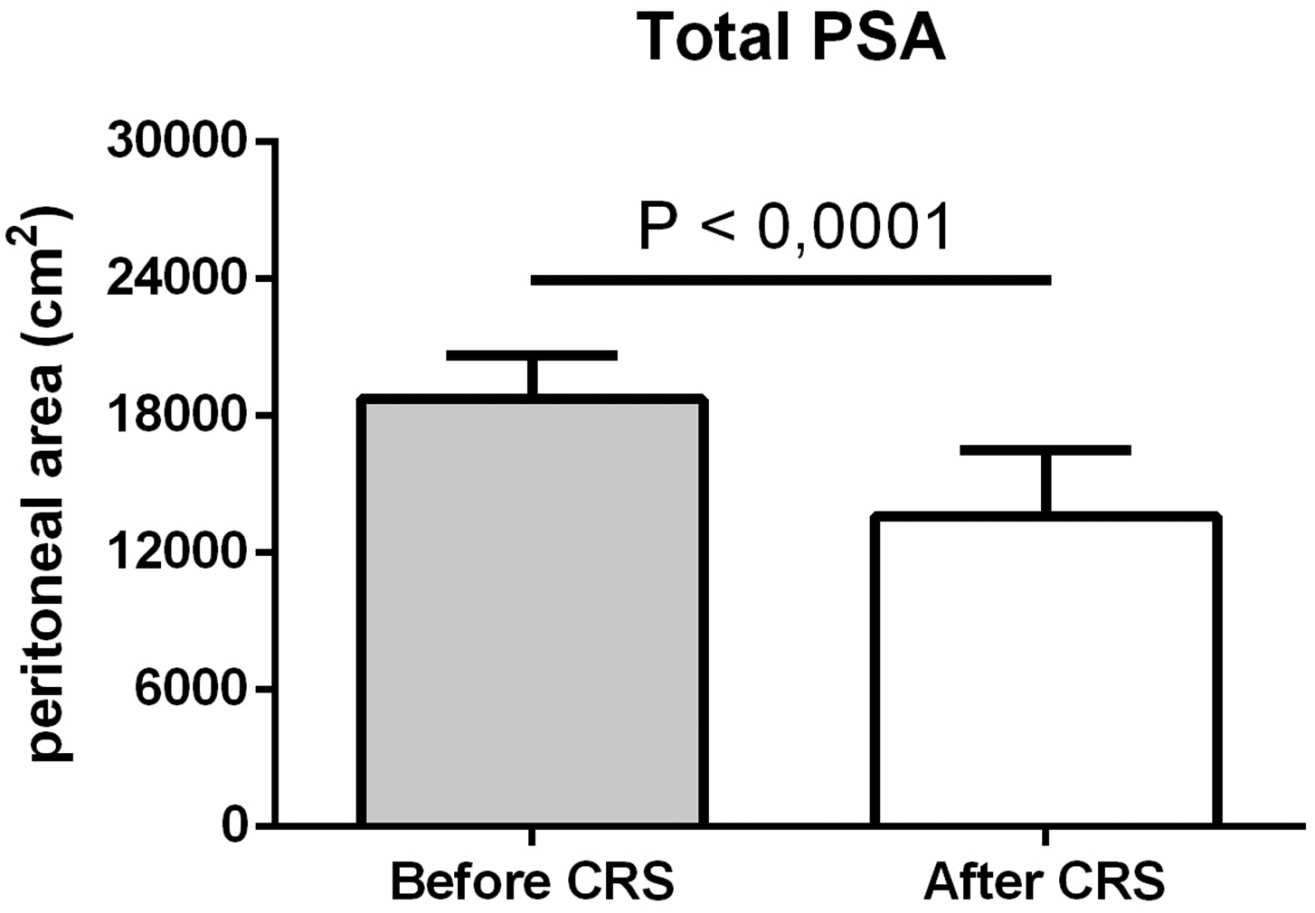
Total peritoneal surface area (PSA) (cm^2^) before and after CRS in 36 patients. Data are shown as mean ± SD.

**Figure 3:**
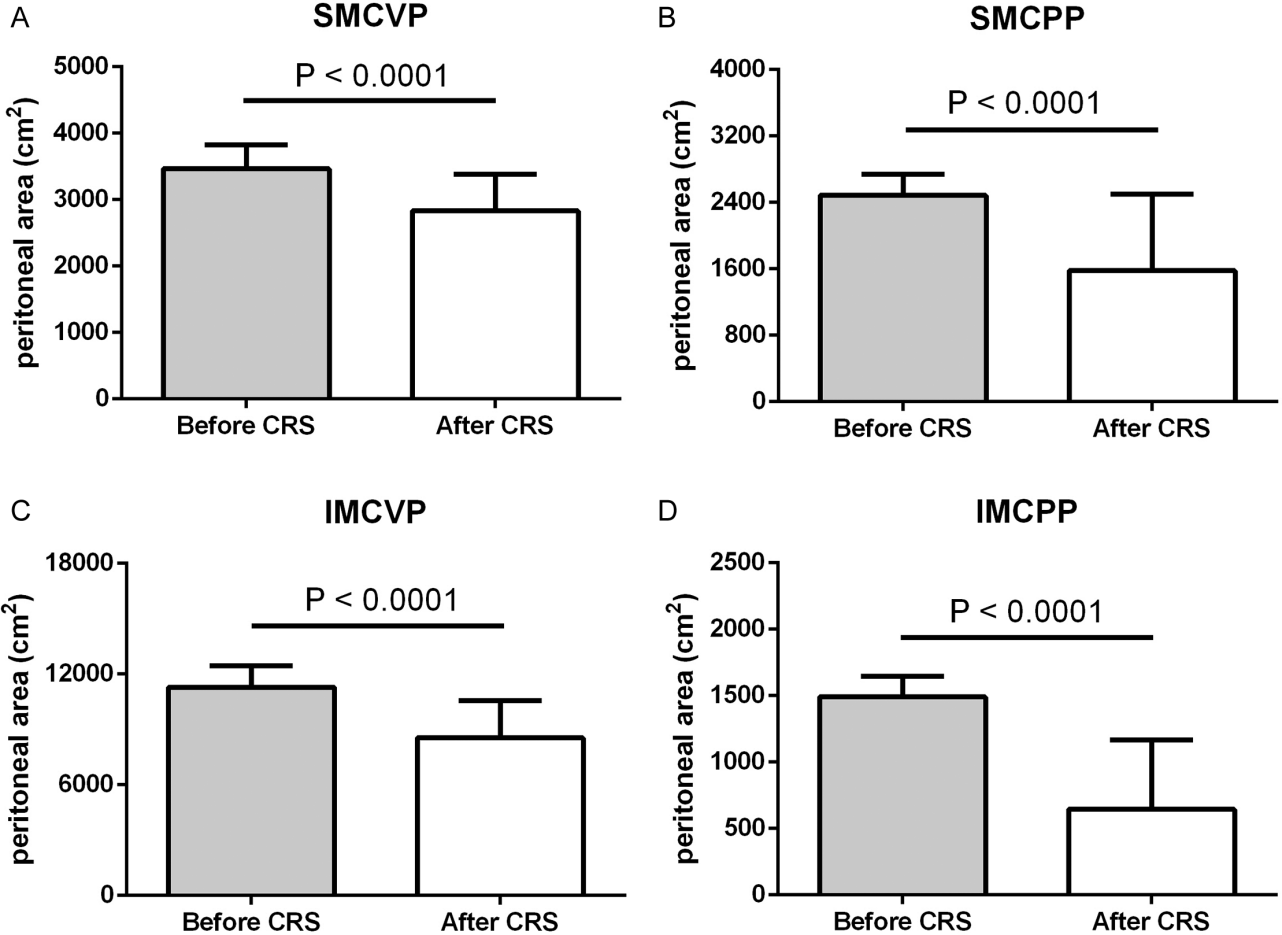
Peritoneal surface areas (cm^2^) before and after CRS in 36 patients in four different anatomical categories. (A) supramesocolic visceral peritoneum (SMCVP), (B) supramesocolic parietal peritoneum (SMCPP), (C) inframesocolic visceral peritoneum (IMCVP) and (D) inframesocolic parietal peritoneum (IMCPP). Data are shown as mean ± SD.

**Figure 4:**
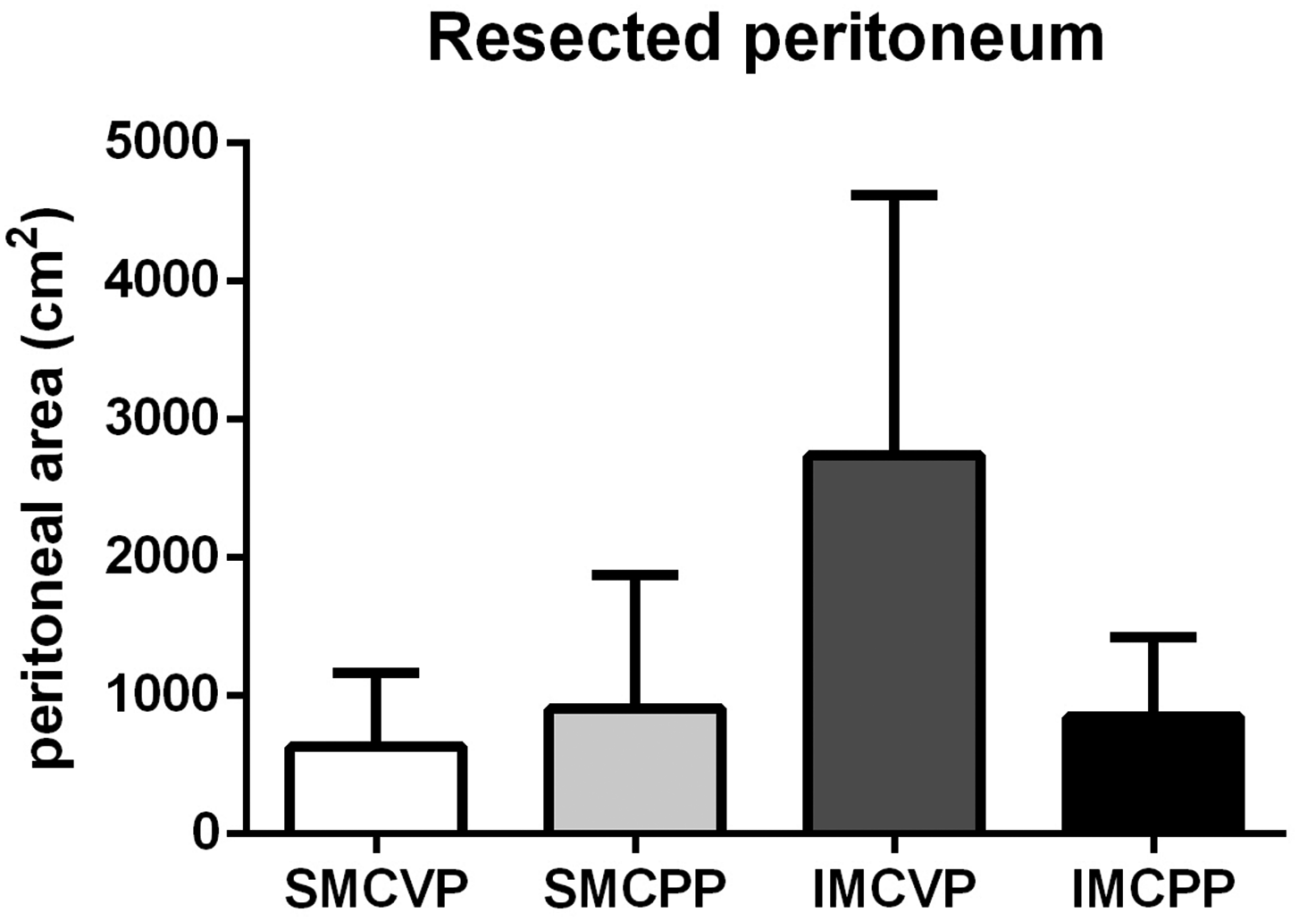
Resected peritoneum (cm^2^) in 36 patients in four different anatomical categories. SMCVP=supramesocolic visceral peritoneum, SMCPP=supramesocolic parietal peritoneum IMCVP=inframesocolic visceral peritoneum, IMCPP=inframesocolic parietal peritoneum. Data are shown as mean ± SD.

**Figure 5:**
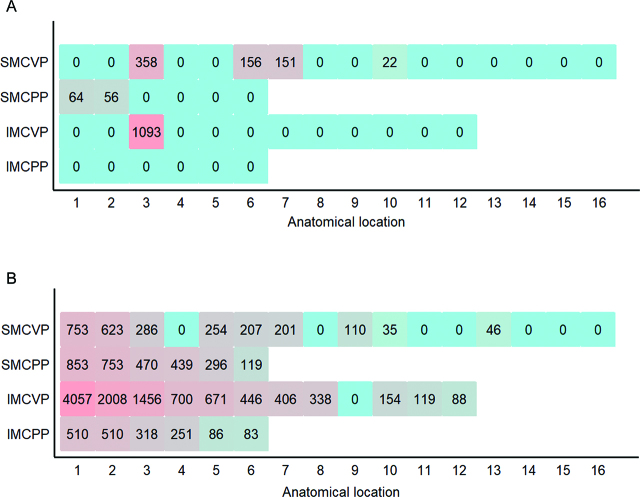
Individual resected peritoneal surface area (cm^2^) heatmaps. (A) One patient who underwent less-extensive peritonectomy and HIPEC is shown. (B) One patient who underwent more extensive peritonectomy is shown. Anatomical region numbers according to Table 1 are labeled on the *x*-axis. SMCVP=supramesocolic visceral peritoneum, SMCPP=supramesocolic parietal peritoneum, IMCVP=inframesocolic visceral peritoneum and IMCPP=inframesocolic parietal peritoneum are labeled on the *y*-axis. Darker colors indicate larger peritoneal surface resections and numbers indicate absolute resected peritoneal surface areas in (cm^2^) calculated by PESUCA.

Patients (n=36) included in the study were mostly affected by peritoneal metastasis of colorectal (n=14), ovarian (n=7), and gastric (n=5) origin. Mean age was 55 years with equal sex distribution. Patients showed a mean peritoneal cancer index (PCI) score of 12. Baseline characteristics are shown in (Table 2). Individual PSA before and after CRS was calculated by PESUCA. The resected PSA of each anatomical region in all patients is shown in Figure 1. The mean PSA of all 36 patients was 18,741 ± 321 cm^2^ before CRS. By entering the peritonectomy extent (%) of each anatomical region (Table 1), PESUCA determined the mean PSA after CRS as 13,611  485 cm^2^ (p<0.0001) (Figure 2). We next analyzed the peritonectomy extent in the four anatomical categories as described in Table 1. The calculated SMCVP area before CRS was 3,464 ± 60 cm^2^ compared to 2,832 ± 92 cm^2^ after CRS (p<0.0001) (Figure 3A). SMCPP area before and after the procedure was 2,485 ± 43 cm^2^ and 1,578 ± 154 cm^2^, respectively (p<0.0001) (Figure 3B). IMCVP area before and after CRS was 11,282 ± 194 and 8,544 ± 336, respectively (p<0.0001) (Figure 3C). IMCPP area before CRS was 1,491 ± 26 cm^2^ compared to 644 ± 87 cm^2^ after CRS (p<0.0001) (Figure 3D). In total, widest peritonectomy extent was performed in the IMCVP area with a mean resected peritoneal surface area (PSA) of 2,738 cm^2^ (Figure 4). The SMCVP area showed lowest peritonectomy extent with a mean resected PSA of 633 cm^2^ (Figure 4). There was a large range of resected PSA, as calculated by PESUCA using the values inputted for 40 different anatomical regions. Therefore, an analysis of the patient with the lowest (1,903 cm^2^) and largest (17,661 cm^2^) resected PSA (Figure 5) was performed.

**Table 2:** Baseline characteristics of 36 CRS and HIPEC procedures.

Characteristics	n (%)
Age, mean ± SD (years)	55 ± 12
Gender	
Female	18 (50%)
Male	18 (50%)
PCI, mean ± SD	12 ± 7
Surgery, mean ± SD (min)	455 ± 132
Chemotherapy	
Oxaliplatin	15 (42%)
Doxorubicin	12 (33%)
Cisplatin	5 (14%)
Mitomycin C	4 (11%)
HIPEC time (min)	
30	15 (42%)
60	18 (50%)
90	3 (8%)
Cancer origin	
Colorectal	14 (39%)
Ovarian	7 (19%)
Gastric	5 (14%)
Mesotheliomas	4 (11%)
Others	6 (17%)
	

HIPEC, hyperthermic intraperitoneal chemotherapy; PCI, peritoneal cancer index.

## Discussion

To the best of our knowledge, here we present the first software solution (PESUCA) to quantify the individual PSA before and after CRS in patients with PSM. CRS combined with HIPEC is a promising therapeutic option for patients with PSM. Its benefit is still controversial even if some encouraging results have been published [4, 12, 13]. The lack of HIPEC procedure standardization could explain contradictory study results [14]. One of the eight parameters influencing HIPEC efficacy is the exact dosing regimen of chemotherapeutic drugs [5, 6]. Sugarbaker et al. [15] assumed that predictions regarding chemotherapy toxicity would be less precise if drug dose and carrier solution volume are not calculated by BSA. Similarly, the COBOX trial showed recently that toxicity and efficacy of concentration based HIPEC protocols in patients suffering from colorectal PSM was higher. It was stated that the concentration-based application is the most standardized way of chemotherapy delivering to the tumor tissue [16]. However, in both current dosing regimens, BSA is used as an estimate of PSA even if CRS has been performed before. The Dedrick formula emphasizes the importance of the effective contact area of the peritoneum and the drug concentration [9]. In patients undergoing CRS with multivisceral resections and peritonectomy procedures the permeability area (PA=effective peritoneal contact area, *A* × permeability, *P*) in the Dedrick formula leads to a lower mass transfer of intraperitoneal chemotherapy into the blood. In contrast, an increased peritoneal surface will result in higher blood levels.

It has been shown that the pharmacokinetics and clearance of intraperitoneal chemotherapy is not affected by the degree of parietal peritoneal resection performed [17], which may be attributed, at least in part, to the fact that the parietal peritoneum only accounts for 20% of the total PSA compared to the visceral peritoneum [10]. Thus, removal of parietal peritoneum has a less-pronounced impact on the permeability area described by the Dedrick formula, than removal of visceral peritoneum. Indeed, it has been described that patients with PSM undergoing large organ resection (resulting in a large reduction in visceral PSA) and HIPEC showed decreased clearance of intraperitoneal chemotherapy [18].

Here, we describe the first tool that provides the ability to quantify the imperfect correlation between actual PSA and calculated BSA in patients undergoing CRS. With our new software, we want to stimulate a discussion regarding the merits of dose adjustment of intraperitoneal chemotherapy during HIPEC based on actual PSA (as calculated using PESUCA during CRS) versus BSA in the context of local chemotherapeutic toxicity. PESUCA considers the decreased permeability area after CRS which influences the chemotherapeutic drug transfer into the blood, and therefore the rate at which the drug can be eliminated from the intraperitoneal cavity. This is not included in both current chemotherapeutic dosing regimens (BSA- and concentration-based). Results calculated by our tool may differ among surgeons performing CRS through variable intraoperative estimations of peritonectomy extent. Further studies are necessary to rule out if a standardized application of PSA calculation by PESUCA in patients undergoing CRS is feasible. After exclusion of peritonectomy estimation bias, our tool should be further investigated to examine if dose adjustments result in less local toxicity by maintaining the same therapeutic effects and thus ensure more patient safety. In addition, PESUCA should be evaluated to determine whether it can function as a new intraoperative classification system and prognostic tool in analogy to the commonly used PCI score. PESUCA is one valuable contribution towards uniform HIPEC standardization, which still presents a major challenge. By establishing more standardization, discrepancies of HIPEC study results could be brought to light and further multicenter randomized controlled trials to rule out real benefits of HIPEC application would be enabled.
